# Development of Iron–Silicate Composites by Waste Glass and Iron or Steel Powders

**DOI:** 10.3390/molecules28176296

**Published:** 2023-08-28

**Authors:** Roxana Rada, Horatiu Vermesan, Simona Rada, Cristian Leostean, Daniela Lucia Manea, Eugen Culea

**Affiliations:** 1 Department of Physics and Chemistry, Technical University of Cluj-Napoca, 400641 Cluj-Napoca, Romania; radaroxananicoleta@yahoo.com (R.R.); horatiu.vermesan@imadd.utcluj.ro (H.V.); daniela.manea@ccm.utcluj.ro (D.L.M.); eugen.culea@phys.utcluj.ro (E.C.); 2 National Institute of Research and Development for Isotopic and Molecular Technologies, 400293 Cluj-Napoca, Romania; cristian.leostean@itim-cj.ro

**Keywords:** iron, steel and glass waste, composites, XRD, IR, UV-Vis, EPR, hysteresis

## Abstract

There is growing interest in the opportunities regarding construction and demolition wastes, such as glass and metal powders, for developing a circular economy and their transformation into new materials. This management and recycling of construction and demolition waste offers environmental benefits and conservation of natural resources. In this paper, new magnetic composite materials were prepared by wet chemical synthesis methods using crushed glasses and iron and steel waste powders as raw materials. The prepared iron–silicate composites were characterized by X-ray diffraction (XRD) and scanning electron microscopy (SEM) analysis, infrared (IR), ultraviolet–visible, and electron paramagnetic resonance (EPR) spectroscopy, and magnetic measurements. The XRD data confirm the formation of varied crystalline phases of the iron ions. The presence of the Fe_3_O_4_ crystalline phase was detected in the composites containing the iron waste powders. The inspection of the SEM micrographs revealed slightly better homogeneity for the composite material containing larger amounts of iron waste and heterogeneous morphology with cracks and random crystallinity for the composite doped with steel waste. By doping with different contents of iron or steel waste powder, structural modifications in the silicate network and the formation of new bands in the IR spectra were evidenced. The UV-Vis spectra were characterized by the absorption peaks for both the tetrahedral and octahedral geometries of the Fe^3+^ ions and the octahedral coordination of the Fe^2+^ ions with oxygen anions. The EPR data show resonance lines with g ~2, 4.3, and 6.4, corresponding to the Fe^3+^ ions. Using hysteresis curves, the superparamagnetic properties of the iron–silicate composites were evidenced.

## 1. Introduction

Construction and demolition waste is composed of concrete, mortar, bricks, ceramics, metals, and glass. Glass is produced at a higher temperature of 1500 °C. In 2018, 130 million tons of glass waste was generated worldwide. Glass recycling can reduce CO_2_ emissions by up to 37% [1].

A large amount of iron powder is generated as waste from the iron, steel, construction, and demolition industries. The accumulation of these residues in the environment has not yet been adequately addressed [2,3]. They are identified as an environmental problem in industrial and civil construction zones. In the context of environmental impact and sustainable development, a significant effort for the utilization of metallic waste has been undertaken across the world. The recycling of iron and steel promotes sustainability, lowering the cost of production, energy usage, and carbon dioxide emissions.

The recycling of waste glasses and metals into new materials is advantageous because significant amounts of raw materials and energy are used in the primary production of these materials. Some authors have recently prepared, using powder metallurgy technique, innovative hybrid nanocomposites using iron waste powder with improved mechanical, tribological, and thermal properties in view of industrial applications [4]. Iron-based catalysts indicate excellent light-to-heat conversion performance and are involved in the conversion of CO_2_ into useful products [5].

The most effective applications are found in the cement and composite industry. Glasses are considered sources of silica in alternative cements and the production of geopolymeric materials as tiles by alkaline activation [6]. Cement paste is subject to a number of modifications with various types of nanoparticles, such as iron oxide and ferrite crystalline phases [7].

Ferrites have captured the attention of scientific communities because of their excellent magnetic, biocompatible, and catalytic properties [8]. These properties are largely dependent on their dimensions, and as a result, the synthesis of uniform nanoparticles is extremely important because, compared to their bulk size, their conductivity and electronic structure change dramatically [9].

Ferrites have technological applications in integrated systems, catalysts, photocatalysts, drug delivery, ferrofluids, sensors, pigments, microwave adsorption, magnetic cores, magnetic recording, shielding and high-frequency devices, and cement composites [3,10,11,12,13].

Nanoferrites are synthesized by various techniques, such as dry and wet chemical synthesis. The microstructure and properties of ferrites are dependent on the different synthesis methods [14]. With current methods, the quality of the nanoparticles is reduced in most cases because a large size distribution is reported and size control is arbitrary [15,16,17]. In most of these methods, a variation in size is achieved only after additional heat treatments of post-synthesis at different temperatures.

The wet chemical synthesis methods, such as sol–gel, co-precipitation and micro-emulsion, have the advantage that the ferrite phases can be obtained only after heat treatments and are accompanied by particle aggregation and growth. The agglomeration of nanoparticles limits the control of the shape, size, and function of the ferrites. Ferrites synthesized by a solid-state reaction at high temperatures (~1200 °C) from oxide and carbonate mixtures have poor compositional control and chemical inhomogeneity [18]. With the continuous improvement of traditional synthesis methods, new synthesis techniques must keep emerging to obtain better performance [19,20].

This work used a wet chemical method without additional heat treatments to synthesize iron–silicate composite materials for specific technological applications using glasses and metal (namely, iron or steel) waste powder as precursors. The prepared materials were characterized by XRD and SEM analysis, IR, UV-Vis and EPR spectroscopy, and magnetic measurements. The aim was to understand the role of alkaline/acidic medium within silicate networks and how the structure of iron–silicate materials is affected by the nature and amount of the iron source.

## 2. Results and Discussion

The data from the literature show that soda–lime glass is most used for the production of windows. Glass waste constitutes SiO_2_ (72–74%), Na_2_O (11–14%), CaO (6–11%), and other oxides, such as MgO, K_2_O, and Fe_2_O_3_ [6,21,22].

### 2.1. XRD Analysis

**Figure 1:**
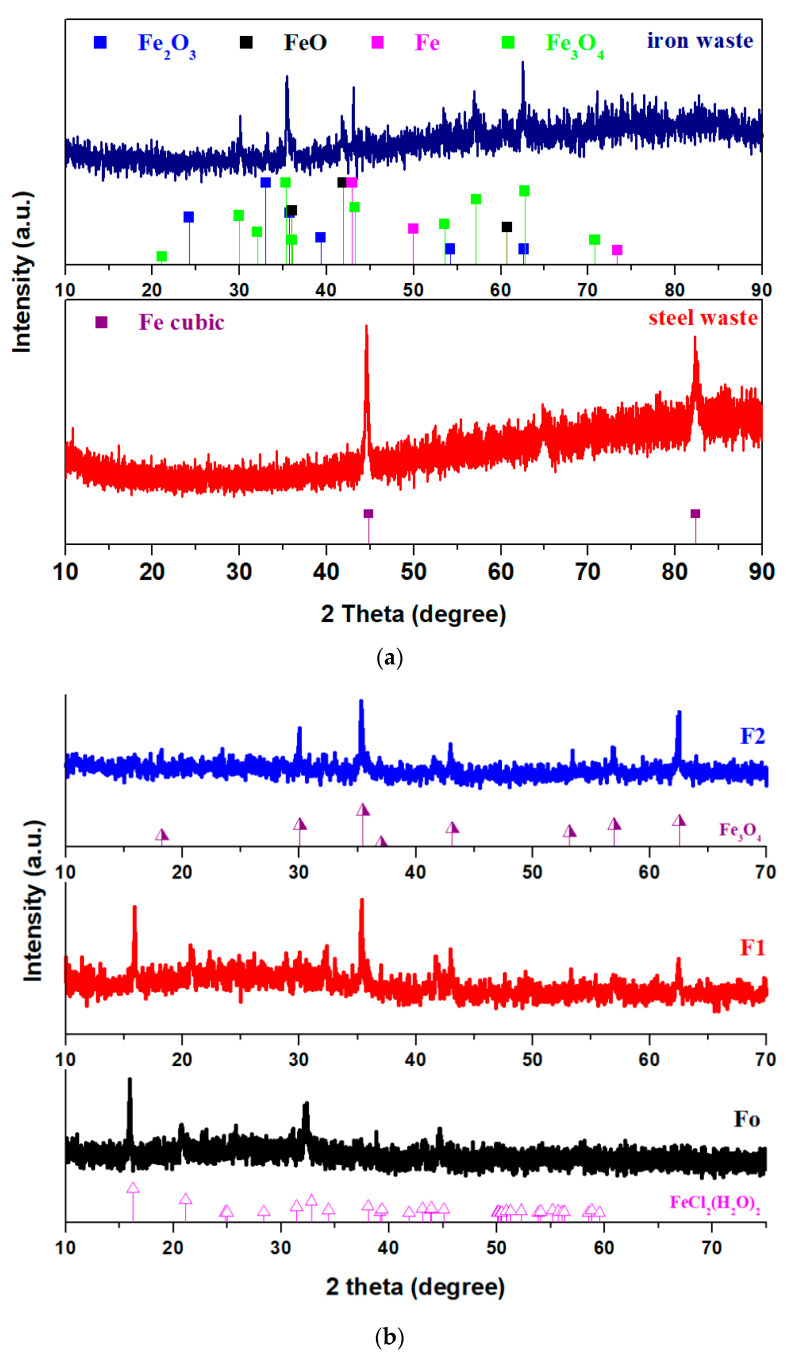
XRD patterns of the (**a**) waste raw materials and (**b**) iron–silicate composites.

X-ray diffractograms of the raw materials (iron and steel waste) and iron–silicate composite materials are shown in Figure 1. The X-ray diffractogram of the steel waste (used as a raw material in this study) shows the majority presence of the iron crystalline phase with a cubic structure. The carbon crystalline phase of the steel waste is below the detection limit of the diffractometer. The pattern of the iron waste shows the same crystalline phases, such as the Fe_2_O_3_ crystalline phase with a rhombohedral structure and Fe, FeO, and Fe_3_O_4_ crystalline phases with a cubic structure. The results indicate that the iron powder was highly oxidated.

The crystalline phases developed in the prepared composite samples depend on the doping nature and the iron content added to the host matrix. For the steel–silica composite, the presence of FeCl_2_·2H_2_O crystalline phase with a monoclinic structure was identified. For the sample with low iron content, the amount of FeCl_2_·2H_2_O crystalline phase decreased, and the formation of Fe_3_O_4_ crystalline phase was confirmed by XRD. At higher iron concentrations, the Fe_3_O_4_ crystalline phase with cubic structure was evidenced as the major phase. The list of peaks and their intensities of the Fe_3_O_4_ and FeCl_2_·2H_2_O crystalline phases are shown in Table 1.

For the iron–silicate composites the formation of varied crystalline phases is dependent on the mass ratio between glasses and iron waste. By increasing iron concentration in the composite structure, the FeCl_2_·2H_2_O (or FeCl_2_(H_2_O)_2_) crystalline phase disappears and the amount of the ferrites phases was enriched.

It is known that Fe_3_O_4_ has a black color. The varied color of the samples and the intensity of the diffraction peaks corresponding to the ferrite phases indicate that the F2 composite has higher ferrite content in the host matrix than its analogs.

The use of this method of synthesis can open new research opportunities in the preparation of ferrites. The oxidized iron waste consisting of varied iron compounds was converted into a Fe_3_O_4_ ferrite crystalline phase by this synthesis method. The advantage of this method without heat treatments consists of the use of precursors as waste powder and 1 M NaOH lower concentration as an alkaline activator. The data from the literature show the compressive strength values of the tiles from waste glass powder are reduced at concentrations above 6M NaOH [6]. An increase in the reactivity of the glassy occurs by adding the acidic medium to the synthesis. The main disadvantages of this study are as follows: (i) the use of powder wastes and (ii) the narrow range of the synthesis temperature for Fe_3_O_4_ ferrites.

### 2.2. Morphology

**Figure 2:**
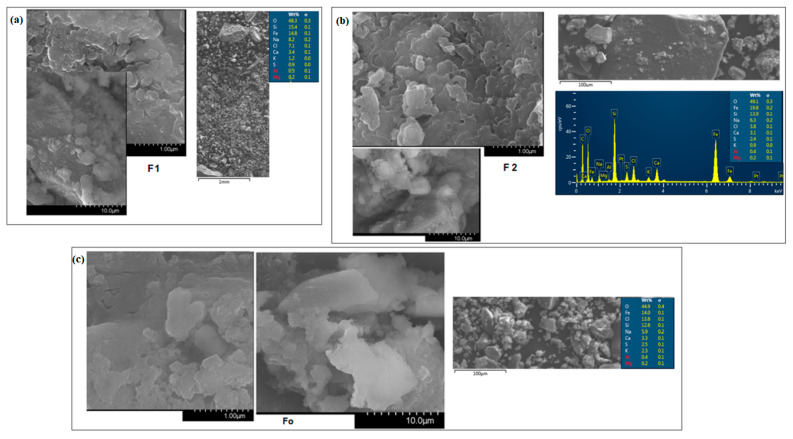
SEM micrographs and EDX analysis of the iron–silicate composite specimens: (**a**) F1, (**b**) F2 and (**c**) Fo samples.

Scanning electron microscopy (SEM) micrographs of the prepared iron–silicate composites are shown in Figure 2. The heterogeneous micrographs with fissures, large structural disorder, and random aggregates with irregular shapes are observed for the steel–iron–silicate Fo composite and iron–silicate F1 composite.

By doping with higher iron content the SEM micrographs show smaller pieces of the network structure, lower structural disorder, and the number of homogeneous grains formed on the surface was increased.

The components contained in composites are investigated using energy-dispersive X-ray (EDX) spectra. EDX analysis of the scanned SEM micrographs of composite samples is given in Figure 2. This analysis shows that the samples consist of oxygen, iron, silicon, sodium, chlorine, calcium, sulfur, potassium, aluminum, and magnesium. The iron content embedded in the host matrix decreases in the following descending order: from 21 weight% for the F2 sample to 14.8 weight% for the F1 sample and 14 weight% for the Fo sample. These results indicate that new products, namely iron–silicate composites, were obtained by the proposed synthesis method.

### 2.3. Structural Investigations by FTIR Spectra

**Figure 3:**
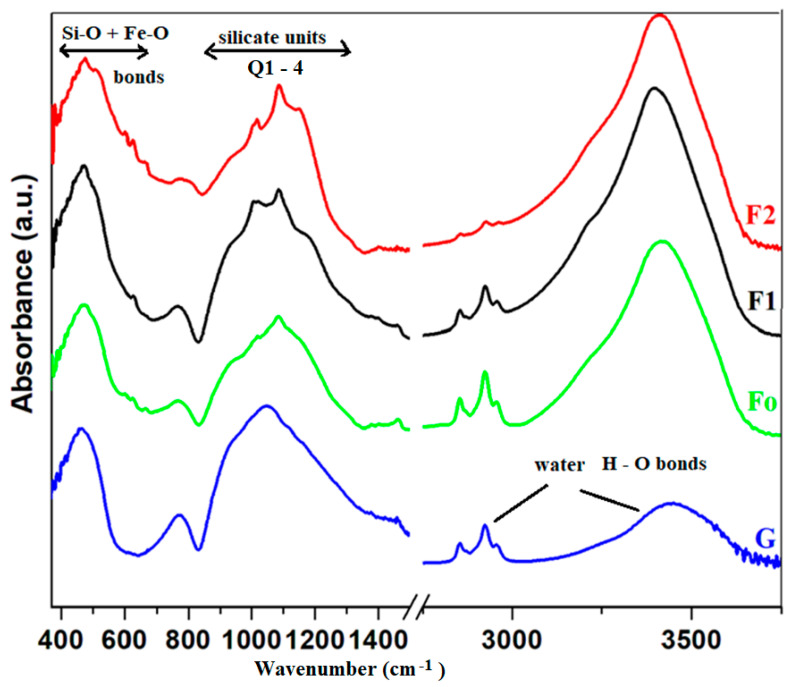
FTIR spectra of the glassy powder and iron–silicate composites.

Infrared spectra of iron–silicate composites are presented in Figure 3. The analysis of IR data indicates three specific domains in the medium frequency region (370–650 cm^−1^), low-intensity bands region (650–850 cm^−1^) and high-intensity bands region (850–1500 cm^−1^). The IR spectra of the silicate matrix display broader IR bands centered at about 470, 770 and 1050 cm^−1^. Specific IR bands of the spinel ferrites appear situated at about 460 and 620 cm^−1^. These bands are related to the stretching vibration of metal–oxygen bonds in octahedral and tetrahedral sites [16,17,23,24].

For the glass waste (G) the IR bands have very broad characteristic features specific to the amorphous structure of the silicate network. A simple inspection of features of the composite samples compared with the silicate host matrix shows a sharp decreasing trend of the strength of the IR bands and also some new shoulders appeared using doping. The Fo and F2 composites have complex structures due to the drastic modifications produced in the region of higher-intensity bands.

The intensity of the IR bands situated between 380 and 600 cm^−1^ was increased by adding iron powder to the host matrix. This increase in intensity can be due to the presence of ferrite phases in the composite. The IR spectra of composite samples differ from the host matrix due to the structural modifications of silicate units (the region between 850 and 1300 cm^−1^) and the presence of ferrite phases (the region between 380 and 600 cm^−1^).

The apparition of an IR band centered at about 615 cm^−1^ can be observed in all composites. The intensity of this band was growth for the F2 sample due to the increase in H_2_SO_4_ volume at synthesis. The fundamental vibration frequencies of sulfate ions occur at ~615 and 1105 cm^−1^ [25].

Magnetite Fe_3_O_4_ (=FeO·Fe_2_O_3_) nanoparticles have two characteristic IR bands situated at about 380 and 570 cm^−1^ corresponding to the Fe-O vibrations modes in the octahedral and tetrahedral geometries. The presence of magnetite nanoparticles can be observed in the IR bands situated at about 570 cm^−1^ which can be shifted to higher wavenumbers with a shoulder [26]. The IR band located at about 380 cm^−1^ shifts towards higher wavenumbers (385 cm^−1^) for the F1 and F2 samples. In our samples, the second band was shifted to higher wavenumbers at 580 cm^−1^ for the F1 composite and 585 cm^−1^ for the F2 composite, respectively, with a shoulder located at about ~625 cm^−1^.

The prominent IR bands situated between 3000–3750 cm^−1^ and the weak IR band located at around 2900 cm^−1^ correspond to the H-O-H bending vibrations and hydroxyl units. The broader IR band centered at about ~3435 cm^−1^ is assigned to the Si-OH stretching [27] and the H-O stretching vibrations from adsorbed water molecules on the surface [28,29,30]. These vibrational frequencies were gradually diminished for the Fo composite.

The F1 and F2 composites differ from the Fo composite in the region of IR bands situated between 375 and 600 cm^−1^. By doping with iron powder this domain is improved while the steel added was decreased. The F2 composite consists of [FeO_4_] and [FeO_6_] structural units while the Fo composite has iron ions bounded with chloride ions, in accordance with XRD data.

For the iron–silicate composites the presence of the IR bands located at about 380 and 530 cm^−1^ are allocated to the Fe_3_O_4_ crystalline phase, in agreement with XRD data. The addition of higher iron content in the host silicate matrix results in a shift at a higher wavenumber of the IR band centered at 470 cm^−1^ and the appearance new IR band situated at about 585 cm^−1^.

IR bands located at about 450 and 680 cm^−1^ may be associated with the deformation vibrations of Si-O-Si angles. The prominent infrared bands (850 and 1300 cm^−1^) are assigned to the stretching vibrations of the Si-O bonds in silicate structural units [31]. The IR band situated at about ~1100 cm^−1^ can be due to the Si-O-Si asymmetric stretching vibrations [27].

The host matrix in the high-frequency region of the IR spectrum is dominated by a strong band centered at about 1050 cm^−1^. The splitting of this band increases and new IR bands appear simultaneously with the addition of higher iron powder. An interpretation of this evolution can be due to the apparition of new silicate units known in the literature as Q1, Q2, Q3 and Q4 structural units. When all four bonds of the silicon atom are formed by bridging oxygens are known as Q4 units and belong to the Q3, Q2 and Q1 units where one, two or three atoms are non-bridging oxygen atoms [30]. The IR bands attributed to Q1, Q2, Q3 and Q4 units are located at about ~930, 980, 1085 and 1135 cm^−1^, respectively, in the IR spectra [27,32]. It can be observed that by the addition of dopant in the host matrix the IR bands corresponding to the Q3 and Q4 units become stronger and the intensities attributed to the Q1 and Q2 units were decreased. By doping, the Q1 and Q2 units are gradually converted to Q3 and Q4 units causing the apparition of more bonding oxygen bonds, and, as a result, the intensity of the Si-OH band (3435 cm^−1^) was increased. The disruption of the silicate network is more accentuated with a higher ratio of iron incorporation in the glass structure causing an improvement in reactivity. The increase in reactivity of the glassy network in the alkaline/acidic medium yields the depolymerization of the silicate network and the appearance of new IR bands corresponding to the varied silicate units [27]. For the F2 composite, an increasing trend of strength of this band occurs and the formation of the Fe_3_O_4_ crystalline phase was evidenced in XRD data.

In brief, the IR spectra of the silicate host matrix and spinel ferrites differ from each other indicating the formation of composites by wet synthesis without heat treatments using as precursors the waste powders, NaOH and HCl solutions (of 1N concentration). When a process of drastic depolymerization of silicate network was produced the intensity of the IR bands situated between 380 and 600 cm^−1^ were increased with the addition of iron powder in the host matrix due to the formation of ferrite nanoparticles. The reactivity of glassy increases in alkaline environments and also in acidic mediums.

The significant difference in the FTIR spectra of the glassy powder and iron–silicate composites can be observed in the structural modifications of silicate units (800–1300 cm^−1^) and the presence of stretching vibrations of Fe-O bond in octahedral and tetrahedral units (380 and 570 cm^−1^). The IR bands shift in frequency and/or intensity by the addition of iron waste in the host network due to the conversion of the silicate matrix in the ferrite into a new silicate matrix.

### 2.4. Structural Investigations of the UV-Vis Spectra

**Figure 4:**
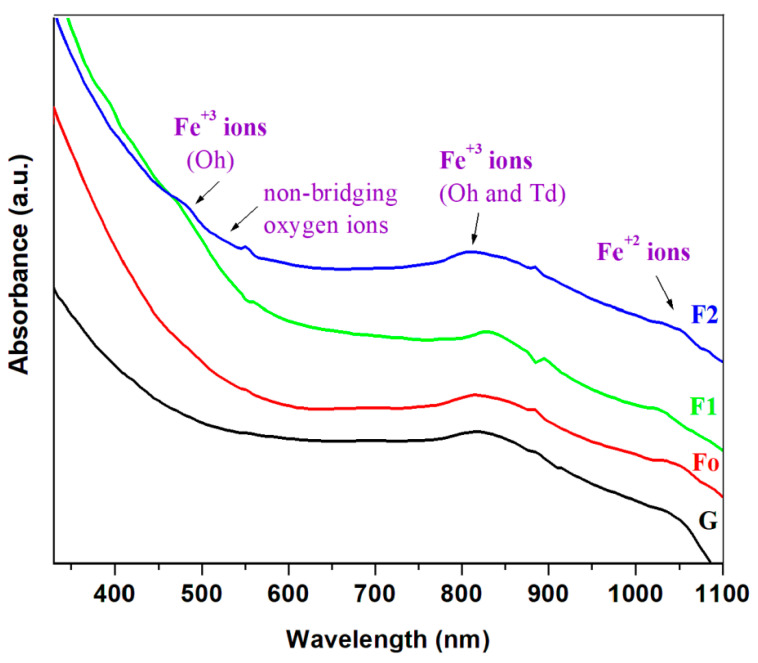
UV-Vis spectra of the iron–silicate composites.

The UV-Vis spectra of the iron–silicate composite materials are displayed in Figure 4. The absorption spectrum consists of some bands related to the electronic transitions of the iron ions. UV-Vis band situated at 460 nm is due to the electronic transitions of Fe^3+^ ions situated in the distorted octahedral (Oh) geometry [33,34] overlapped with non-bridging oxygen ions. For the samples doped with steel content, an increasing trend of the position of the edge towards a higher wavelength can be associated with the increase in non-bonding oxygen ions and Fe^3+^ ions.

The UV-Vis bands located in the region between 850 and 1050 nm are assigned to the d-d electronic transitions of t_2_g → e_g_ type in the octahedral geometry of the Fe^2+^ ions [35].

The UV-Vis bands situated at about 585 and 785 nm were attributed to the electronic transitions of Fe^3+^ ions situated in the octahedral (Oh) and tetrahedral (Td) symmetry with oxygen atoms. The intensity of these bands reaches the maximum value in the F1 and F2 composites. The increase in intensity of the UV-Vis bands centered at about 400 and 1050 nm indicates the presence of Fe^2+^ and Fe^3+^ ions and the formation of the Fe_3_O_4_ crystalline phase (Fe_3_O_4_ = Fe^2+^ O·Fe_2_^3+^O_3_).

In brief, UV-Vis spectra evidence the presence of Fe^2+^ and Fe^3+^ ions situated in [FeO_4_] tetrahedral and [FeO_6_] octahedral units. The coordination chemistry shows the accommodation with [FeO_4_] units for Fe^2+^ ions and with [FeO_6_] units for Fe^3+^ ions. By doping with higher iron content a conversion process of Q1 and Q2 units into Q3 and Q4 units happened in accordance with IR data. The mutual concentration of Fe^2+^/Fe^3+^ ions depends on synthesis conditions and on the silicate conversion process which results in the formation of non-bridging oxygen ions. The excess of non-bridging oxygen ions will be coordinated with Fe^2+^ and Fe^3+^ions.

### 2.5. Band Gap Energy

**Figure 5:**
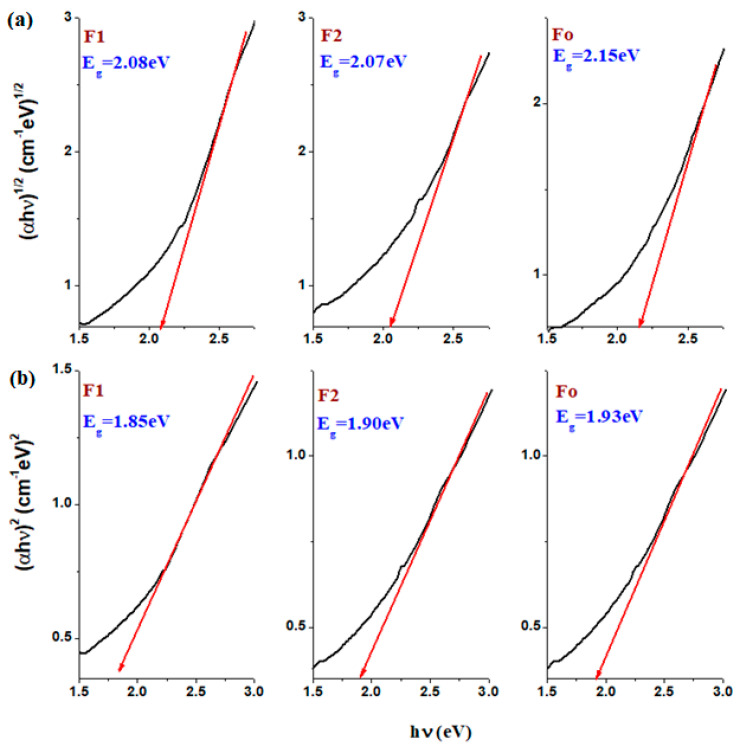
The (**a**) (αhν)^2^ and (**b**) (αhν)^1/2^ versus the photon energy (hν) for the studied composites. The red line shows the extrapolation of the gap energy.

Figure 5 shows the plots *(αhν)^n^* versus *hν* of the *n* = 2 and *n* = ½ which are used to estimate the band gap energies. The values of the optical band gap energies, *E_g_* were estimated from the extrapolating of the tangent linear fit of these curves corresponding to the absorption edge range. The detailed knowledge regarding the optical band gap, the direct and indirect transitions and the tangent line of the plots which make the estimations of *E_g_* values in the disordered systems were shown in the references [36,37,38,39,40,41,42].

The band gap energy values lie between 1.85 and 2.15 eV. The error of the estimated *E_g_* values can be between 0.5 and 1 eV [36].

The decrease in gap energy value with the addition of iron content in the host matrix suggests the formation of a larger number of defects and non-bonding oxygen ions which affects the structural properties. For the doping with steel of the silicate glassy the concentration of the non-bridging oxygen ions decreases which results in an increase in the band gap energy.

The narrow band energy gap (~2 eV) makes super-paramagnetic ferrites as potential materials to degrade dyes and organic contaminants because can realize the fast separation of photo-induced electron–hole pairs under visible light [42,43].

### 2.6. Structural Investigations of the Electron Paramagnetic Resonance (EPR) Data

**Figure 6:**
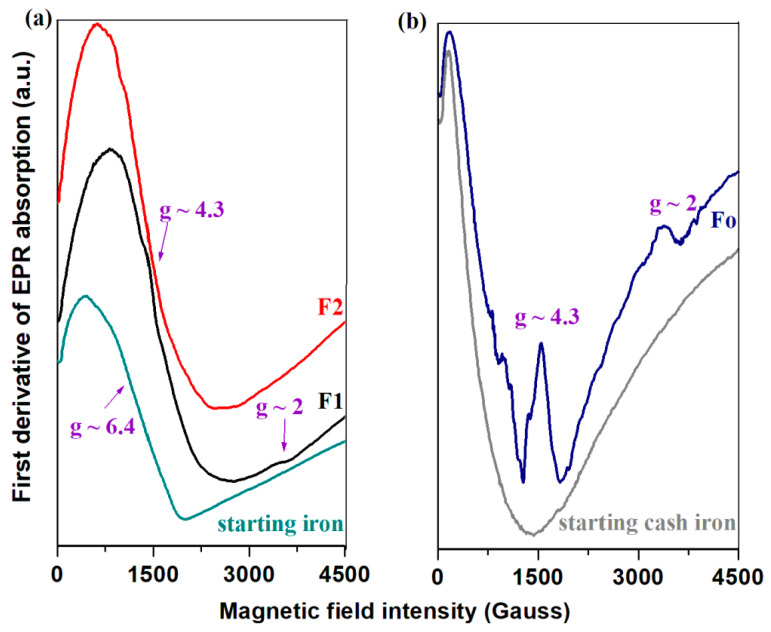
EPR spectra of (**a**) the starting iron powder and iron–silicate composites; (**b**) the starting steel powders and steel–silicate composite.

Figure 6 shows EPR spectra for the starting materials and prepared composites. At room temperature, the EPR spectra of Fe^3+^ ions are characterized by the appearance of resonance lines located at g ~2, 4.3 and 6.4 [44,45,46,47].

The iron waste used in this study shows two resonance signals situated at about g ~4.3 and 6.4. The EPR parameters show the presence of Fe^3+^ ions in this powder originated from oxidized iron, in agreement with XRD data. For the steel waste, the absence of Fe^3+^ ions was evidenced in the EPR spectrum. For the F1 and F2 composites the apparition of Fe_3_O_4_ ferrite phases in the host matrix can be attributed to the existence of Fe^3+^ ions in the raw material (iron powder).

The recorded EPR spectra of the prepared composites exhibit intense resonance signals near g~4.3 and weak resonance lines centered at about g ~2 and 6.4. The resonance signal located at about g~2 is due to the clustered Fe^3+^ ions which interact by the super-exchange or dipole–dipole interactions. The feature with g~4.3 arises from the isolated Fe^3+^ ions situated in rhombic distorted octahedral or tetrahedral geometries and with g~6.4 originates from axially distorted geometry.

For the F2 composite, the feature centered at about g~2 attributed to the interacting Fe^3+^ centers disappears and the resonance line located at about g~6.4 becomes more prominent. In the F1 sample, an increase in the intensity of the resonance lines situated near g ~4.3 and 6.4 was evidenced. With the addition of steel in the host network the intensity of resonance lines located at about g ~2, 4.3 and 6.4 decrease drastically, and for the signal located at about g~4.3 the presence of hyperfine structure is also observed.

For the Fo and F1 composites, the resonance line with g~4.3 decreases due to the growth of the Fe^2+^Cl_2_(H_2_O)_2_ crystalline phase in accordance with XRD data. The amount of Fe^2+^ ions is higher than that of Fe^3+^ ions due to the Fe^3+^ ↔ Fe^2+^ conversion. By increasing iron levels in the F2 composite, an opposite effect can be observed due to the disappearance of iron (II) crystallites.

In the F2 sample, the amount of Fe^3+^ ions and the process of depolymerization of the silicate network were increased, and, as a result, the amount of the Fe_3_O_4_ crystalline phase was enriched.

### 2.7. Hysteresis

**Figure 7:**
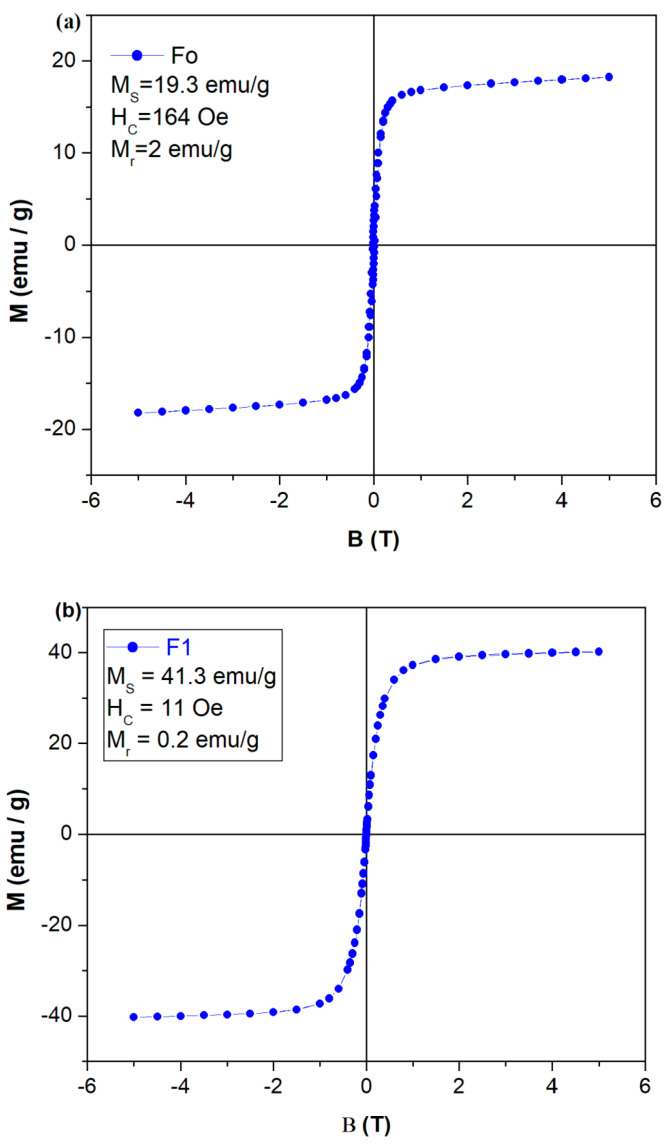

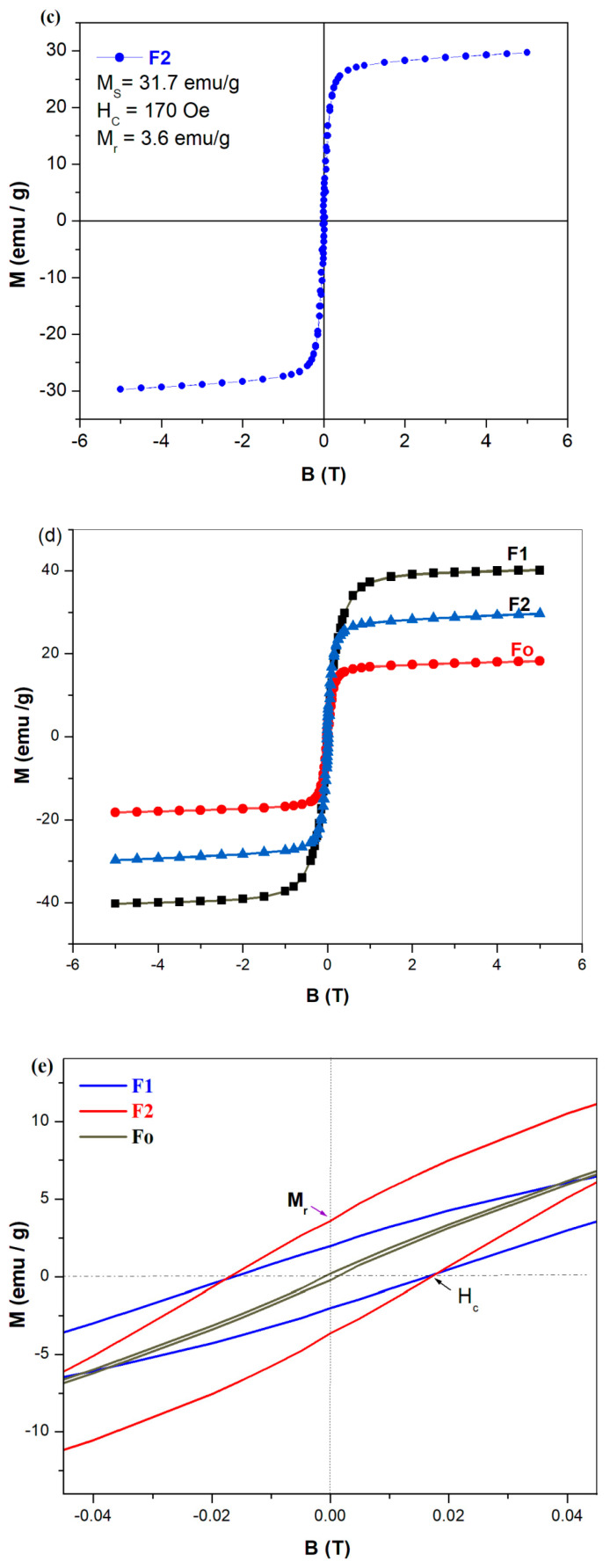
Hysteresis loops of doped silicate composites: (**a**) Fo sample, (**b**) F1 sample, (**c**) F2 sample, (**d**) Fo, F1 and F2 samples; (**e**) a zoom of hysteresis curves with the data close to the 0, 0 region.

The hysteresis loop can be related to the existence of a magnetic domain in ferromagnetic samples and to determine spins in magnetic materials. The magnetic hysteresis curve will be used to identify the type as hard, soft and super-paramagnetic ferrite. Figure 7 shows the hysteresis curve of the iron–silicate composites at room temperature. The dependence of magnetization, M (emu/g) as a function of the applied magnetic field of the doped silicate composites shows that the nanocomposites have soft-ferromagnetic behavior [48]. The first direct observation of the hysteresis loops is that the saturation magnetization, M_S_ of F1, and F2 composites are higher than that of the Fo composite. At smaller iron contents in the host matrix, the saturation magnetization is lower in the hysteresis curve.

Data from the literature show the values of saturation magnetization for the Fe_3_O_4_ nanoparticles situated between 80 and 98 emu/g, respectively. The saturation magnetization of doped silicate composites, for example, (Mn_0.5_Fe_0.5_)_2_SiO_4_ is 4.9 emu/g [49], and has a lower value than that of iron–silicate composites. The effect of variation of FeCl_2_ concentration on the magnetic properties of the nanoparticles shows that the value of saturation magnetization decreases about 30–40% of the bulk material [50].

The Fe_3_O_4_ nanoparticles have a higher magnetization (between 80 and 98 emu/g) and for our samples, the saturation magnetization was reduced to 41.3 emu/g for sample F1, at 31.7 emu/g, for the sample F2 and 19.3 emu/g for the sample Fo, respectively, due to the non-magnetic silicate network [51]. For the Fo sample lower value of the saturation magnetization (19.3 emu/g) was evidenced due to the presence of FeCl_2_(H_2_O)_2_ crystalline phase.

The small change in saturation magnetization can be due to the modification in the particle sizes of the materials [52]. The F2 composite indicates smaller particle sizes in comparison to the F1 composite, in accordance with SEM data.

The values of coercivities, H_C_ of the Fo and F2 composites, are significantly larger than that of the F1 sample and are associated with the pronounced ferromagnetic order.

**Figure 8:**
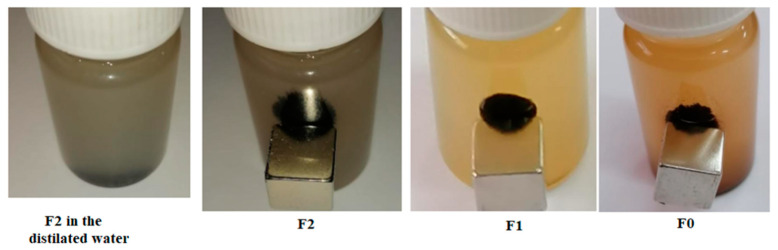
Dispersion and separation behaviors in the water of the prepared composite materials.

The magnetic properties of the synthesized samples were evaluated by the dispersion and separation behaviors in distilled water. The dispersion and separation behaviors of the composites in distilled water using a neodymium N42 magnet are presented in Figure 8. The behaviors of the samples demonstrate the dispersion and rapid magnetic receptivity when a magnetic field is applied. The aqueous solubility of the composites shows a dispersible non-magnetic part and a heavy black magnetic part. Furthermore, for sample F1, the remanance magnetism, Mr, and coercive force, Hc were obtained as 0.2 emu/g and 11 Oe, respectively. The lack of a hysteresis loop and the small amounts of remanance and coercivity confirm that the Fe_3_O_4_ nanoparticles go toward super-paramagnetic features which means that they can retain or resist the magnetization after the removal of the magnetic field. The prepared sample had acceptable magnetic responsiveness for the magnetic separation of the solid catalyst. Our results evidence the possible applications in the manufacturing of abrasive materials and the field of photo-catalysis [12,21].

## 3. Experimental Procedure

All the starting chemicals were commercially available, namely natrium hydroxide (NaOH, Sigma-Aldrich, St. Louis, MO, USA, 98%), sulfuric acid (H_2_SO_4_, Emsure, St. Louis, MO, USA, 95–95%), and chlorhydric acid (HCl, Emsure, 37%). The chemicals and waste quantities are listed in Table 2.

In a ceramic crucible the waste glass powder (noted with G) from the broken window was mixed in the first step with NaOH solution of 1N concentration for 10 min at 40 °C. Sodium hydroxide solution of 1N concentration was added to the mixture as alkaline activator. When the glass content was partially dissolved, 5ml of 1N HCl solution was added. Waste iron or steel powders were treated with an H_2_SO_4_ solution of 10% concentration and after that were added to the mixture. The resultant mixture was also stirred for 30 min at 40–50 °C. The temperature was raised to 100 °C (for 10 min) and, finally, to 250 °C. The final solid sample was cooled to room temperature.

The color of the samples depends on the dopant nature and the iron content. The sample containing steel is brown while the samples containing iron are colored gray (for sample F1) or black (for sample F2).

The prepared materials were investigated by analysis of X-ray diffraction using an XRD-6000 Shimadzu diffractometer. The FT-IR spectra of the samples were obtained in the 350–1500 cm^−1^ spectral range with a JASCO FTIR 6200 spectrometer using the standard KBr pellet disc technique. UV–Visible absorption spectra of the powdered samples were recorded at room temperature in the 300–1100 nm range using a Perkin–Elmer Lambda 45 UV/VIS spectrometer equipped with an integrating sphere. EPR measurements of the powder samples were performed with an ADANI spectrometer in X-band. The magnetic measurements were performed at room temperature using a Vibrating Sample Magnetometer.

## 4. Conclusions

In this paper, three composites were prepared to use as raw materials for construction and demolition waste, namely broken windows, oxidized iron and steel powder. Synthesis, characterization and magnetic properties of iron–silicate composites were reported. XRD patterns show the formation of Fe_3_O_4_ crystalline phase by doping with iron waste. The IR data show the depolymerization of the silicate matrix and the presence of stretching vibrations of the M-O bonds in the octahedral and tetrahedral sites from ferrite, by doping with iron waste.

UV-Vis spectra evidence the presence of iron ions in different oxidation numbers (Fe^2+^, Fe^3+^ ions) and with varied coordination geometries ([FeO_4_] tetrahedral and [FeO_6_] octahedral units). By increasing iron content in the silicate network, the number of Fe^3+^ ions was improved, in accordance with UV-Vis and EPR data.

The EPR spectra are typical for the paramagnetic Fe^3+^ ions with the signals centered at about g ~2, 4.3 and 6.4. By doping with steel and smaller iron content, the intensities of the last resonance lines were diminished suggesting a decrease in isolated distorted geometries. The value of the saturation magnetization depends on the crystalline phases of the iron ions developed in the silicate network. The saturation magnetization of the F1 composite was 41.3 emu/g after that its value decreased to 31.7 emu/g in the F2 composite for higher iron content in the host network.

## Figures and Tables

**Table 1:** Identification phase from powder diffraction: list of peaks and their intensity of the Fe_3_O_4_ and FeCl_2_(H_2_O)_2_ crystalline phases.

FeO·Fe_2_O_3_ = Fe_3_O_4_ (Magnetite) with Cubic Structure		FeCl_2_(H_2_O)_2_ with Monoclinic Structure	
2 Theta (Degree)	Intensity (%)	2 Theta (Degree)	Intensity (%)
18.24 30.06 35.45 37.04 43.10 53.14 56.98 62.54 71.03 74.07	30 60 100 10 50 40 60 70 10 30	15.98 20.77 24.43 24.71 28.03 31.03 32.37 32.49 33.75 37.52 38.53 38.77 41.17 42.26 43.17 43.27 43.37 49.31	100 57.35 1.4 2.6 3 25.02 47.64 29.52 11.31 27.12 3.9 14.41 1.2 16.31 13.01 18.31 14.01 2.8

**Table 2:** Summary of the quantities of the reagents and waste powders.

Name	Sample Code	Waste Glass Powder (g)	HCl (mL)	NaOH (mL)	H_2_SO_4_ (mL)	Waste Cast Iron Powder (g)	Waste Iron Powder (g)
Steel–silicate composite	Fo	5	5	5	2	5	-
Iron–silicate composite	F1	5	5	5	2	-	5
Iron–silicate composite	F2	5	5	5	5	-	7.5

## Data Availability

Not applicable.

## Sample Availability

Samples of the iron–silicated compounds are available from the authors.
